# The fungal pathogen *Batrachochytrium dendrobatidis* drives the relationship between environmental and amphibian skin microbiota

**DOI:** 10.1093/ismeco/ycag016

**Published:** 2026-02-04

**Authors:** Rayan Bouchali, Hugo Sentenac, Dirk S Schmeller, Adriana Bernardo-Cravo, Adeline Loyau

**Affiliations:** Université de Toulouse, Toulouse INP – Institut National Polytechnique de Toulouse (National Polytechnic Institute of Toulouse), CNRS – Centre National de la Recherche Scientifique (National Center for Scientific Research), IRD – Institut de Recherche pour le Développement (Research Institute for Development) CRBE – Centre de Recherches sur la Biodiversité et l'Environnement, Toulouse, France; Université Marie et Louis Pasteur, CNRS, Chrono-environnement (UMR 6249), F-25000 Besançon, France; Université de Toulouse, Toulouse INP – Institut National Polytechnique de Toulouse (National Polytechnic Institute of Toulouse), CNRS – Centre National de la Recherche Scientifique (National Center for Scientific Research), IRD – Institut de Recherche pour le Développement (Research Institute for Development) CRBE – Centre de Recherches sur la Biodiversité et l'Environnement, Toulouse, France; Université de Toulouse, Toulouse INP – Institut National Polytechnique de Toulouse (National Polytechnic Institute of Toulouse), CNRS – Centre National de la Recherche Scientifique (National Center for Scientific Research), IRD – Institut de Recherche pour le Développement (Research Institute for Development) CRBE – Centre de Recherches sur la Biodiversité et l'Environnement, Toulouse, France; Université de Toulouse, Toulouse INP – Institut National Polytechnique de Toulouse (National Polytechnic Institute of Toulouse), CNRS – Centre National de la Recherche Scientifique (National Center for Scientific Research), IRD – Institut de Recherche pour le Développement (Research Institute for Development) CRBE – Centre de Recherches sur la Biodiversité et l'Environnement, Toulouse, France

**Keywords:** alpine lakes, chytrid fungus, microbiota, disease ecology, microbial coalescence, amphibian immunity

## Abstract

Microbial coalescence is a key process driving the assembly of communities when diverse compartments of ecosystems meet. Coalescence is likely involved in structuring amphibian skin microbiota, which play a crucial role in host immunity, but whose environmental microbial sources remain unknown. Here, we investigated the microbial sources (water and biofilm microbiota, i.e. adherent microbial community embedded collectively on submerged rocks) and coalescence processes of the skin microbiota of three amphibian species (*Alytes obstetricans*, *Rana temporaria*, and *Bufo spinosus*), in 20 mountain lakes of the French Pyrenees, infected or not with the fungal pathogen *Batrachochytrium dendrobatidis* (*Bd*). We used 16S ribosomal ribonucleic acid gene metabarcoding coupled with a Bayesian SourceTracker analysis and a phylogenetic null model. We found that the amphibian skin microbiome originated mainly from environmental water (9%–23%), less from biofilm (3%–6%), and not from horizontal transfer. Host exposure to *Bd* strongly influenced microbial engraftment. The presence of the pathogen probably did not affect the number of bacterial taxa shared between environmental and skin microbiotas, but enriched some of them, including protective ones, from the water only. Stochastic processes dominated the structuration of the resulting communities, but some deterministic selection probably occurred, maybe via microbiome dysbiosis that favor higher abundance of anti-*Bd* genera, which often are environmental opportunists. Our study provides first insights into the importance of microbial coalescence in shaping the amphibian skin microbiome, and the role of environmental microbial communities in mounting disease resistance.

## Introduction

Microorganisms are everywhere, organized in highly dynamic communities that are in constant exchange with the environment, other living organisms, and each other. However, we have limited understanding on the importance of encounters between microorganisms from different ecosystem compartments in the shaping processes of microbial communities. Microbe transfers and their fate have only recently been conceptualized under the term of “microbial community coalescence” [1, 2], which takes place in two successive steps. In a first step, that we call “microbial transfers”, microbes are exchanged between compartments, mostly stochastically, by wind, water flow, animal burrow, grazing, or feces spillover [3, 4]. The second step of microbial coalescence consists in the assembly of the final community, known as the “engrafted community”. The engrafted community results from ecological interactions between initial community members, newly transferred microbes, and selective processes such as priority effects, environmental filtering, ecological interactions, and top-down co-selection [2, 5, 6]. Microbial coalescence is particularly relevant in the global change context, which promotes and increases connections and encounters between biological systems, and could be involved in pathogen spillovers and habitat invasibility [2, 7, 8].

Despite the growing recognition of coalescence in microbial ecology, its implications for host-associated microbiota remain largely unexplored. Only a few studies have demonstrated the role played by environmental microbial communities in shaping human and animal microbiota through coalescence processes. Mice exposed to new microbial cells tend to favor the microorganisms best suited to utilizing host energy sources [1]. Mixing between human oral and fecal bacterial communities promotes high rates of *Streptococcus* and *Stenotrophomonas* that could lead to colon cancer or liver cirrhosis [9]. Persistent changes in human skin microbiota have been observed following contact with soil or plant leaves, and transfers were found stronger when coming from high-biomass sources [10, 11]. Studies focusing on these processes in the natural environment are even scarcer [2, 4]. Thus, how environmental microbial communities and other potential sources of microorganisms contribute to the composition and functional properties of host microbiota remains poorly understood, as do the assembly processes at work.

Amphibians emerge as an appealing model system given the large number of studies focusing on their skin microbiota [12] and its relevance in the context of worldwide declines of amphibians due to the lethal fungal pathogen *Batrachochytrium dendrobatidis* (*Bd*), responsible for the skin disease chytridiomycosis [13]. The amphibian skin microbiota plays a major role in immunity against *Bd* [14, 15]. Amphibians with skin microbiota having high rates of the bacteria *Janthinobacterium lividum* showed lower morbidity and mortality due to chytridiomycosis [16, 17]. More broadly, *Pseudomonas*, *Bacillus,* and *Chitinophaga* isolates from the Red-backed salamander also demonstrated protective capacities against *Bd* [18]. Bacterial combinations were shown to be more effective in combating the pathogen than the same bacteria alone, underlining the importance of microbial communities in amphibian immunity [19].

A better comprehension of the shaping processes of the amphibian skin microbiota, as well as its functional implications, are therefore necessary steps toward the prevention of amphibian declines [13, 18, 20–22]. In an experimental setting, amphibian skin microbiome of tadpoles was found to be seeded by the foam-nest, the surrounding water, as well as the parental microbial communities, but a large proportion came from unknown sources [23]. While there are very few studies in natural settings, amphibian skin microbiota may come from several, non-mutually exclusive, sources: (i) the parental microbiota (vertical transfer [24], (ii) conspecifics and other members of the amphibian community (horizontal transfer [25, 26]), and/or (iii) the surrounding environmental microbial communities (e.g. from sediments, soil, water, and biofilms [23, 27]). Amphibian skin is not only colonized by microorganisms from the environment, but also serves as a site for reassembly and selection of specific cells [28], including the recruitment of rare environmental bacteria [29], and/or enrichment of beneficial bacteria helping to combat perturbations and pathogens [15]. For example, Loyau and colleagues [30] observed that hosts sharing the same habitat had significant differences in the relative abundance of putative *Bd*-inhibitory bacteria linked to their susceptibility to *Bd*. Protective bacteria were more frequent in *Bufo spinosus* and *Rana temporaria* in comparison to *Alytes obstetricans*, the species most susceptible to *Bd* chytridiomycosis in this system [30]. Moreover, amphibian skin microbiota sampled from lakes having populations in which *Bd* infections were detected (hereafter, *Bd*-positive lakes) displayed higher levels of putative anti-*Bd* bacteria. This change was associated to different bacterial communities between amphibians from infected and non-infected populations [30]. This suggests differentiated building processes according to the host species and the presence of the pathogen, which could help mitigate infection severity [27, 31].

To test the hypothesis of a differentiated amphibian skin building processes in presence of *Bd*, we used 16S ribosomal ribonucleic acid (rRNA) metabarcoding approach on samples from 20 high altitude mountain lakes across the French Pyrenees (Europe) and during a three-year period. We considered three amphibian species, *A. obstetricans*, *B. spinosus*, and *R. temporaria*, whose populations were infected or not by *Bd*. We quantified the microbial transfers occurring from the environmental microbial communities (from biofilms and water) to the skin microbiota of amphibian tadpoles using a Bayesian SourceTracker analysis. A phylogenetic null model was used to investigate if the microbial communities engrafted on tadpole skin were shaped more by deterministic (e.g. selection by the host) or stochastic (e.g. passive dispersal) processes. Finally, we tested if the presence of *Bd* in the environment altered microbial transfer between sources and amphibian tadpole skin, including for putative anti-*Bd* bacteria, also if *Bd* impact the assembly processes of the engrafted communities.

## Material and methods

### Study sites, monitoring of environmental data, and sampling strategy

**Figure 1:**
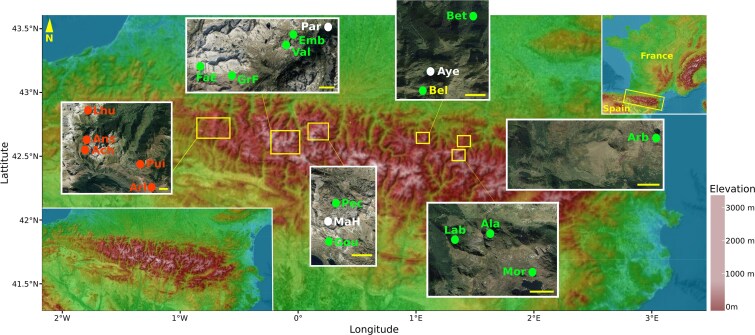
Location of the 20 lakes in which biofilm, water and amphibian skin microbiota were sampled. Yellow scale bars represent 1 kilometer. Lakes indicated in green are considered *Bd*-negative, those in red are considered *Bd*-positive. Lakes with an unclear infectious status are showed in white. Abbreviations of lake names are: Lhu = Lhurs, Ans = Ansabère, Ach = Acherito, Pui = Puits d’Arrious, Arl = Arlet, FaE = Fache-Espagne, GrF = Grande-Fache, Val = Vallon, Emb = Embarrat, Par = Paradis, Pec = Pêcheur, MaH = Madamète-Haut, Gou = Gourg-de-Rabas, Bel = Bellonguère, Aye = Ayès, Bet = Bethmale, Lab = Labant, Ala = Alate, Mor = Mort and Arb = Arbu. Elevation map modified from https://fr-fr.topographic-map.com. Satellite view from Google Earth©.

Twenty mountain lakes were sampled for water, biofilms developed on submerged rocks, and amphibian tadpole skin microbiota. All lakes are located in the Pyrenean mountains (France, Europe) (Table 1; Fig. 1). Our lake system has been monitored for over 20 years for the presence of *Bd* [32–34]. For the years covered by our study, we confirmed the presence of *Bd* by performing a duplicate quantitative polymerase chain reaction on amphibian tadpole skin samples as described in [35] (see Table S1 for prevalence and *Bd* loads). The detection of *Bd* on amphibian tadpole skin allowed us to characterize five *Bd*-positive lakes (*Bd* detected every year). Twelve lakes were considered as *Bd*-negative, and three lakes had a changing infection status (Table 1; Fig. 1). The sites were visited several times during early, high or late summer, two times in 2016, three times in 2017 and 2018. Water from the littoral zone was collected on each lake using a sterile bottle thoroughly rinsed with lake water. Between 250 ml and 1 liter of the collected water were filtered on a 0.22 μm mesh using a vacuum pump. Biofilms were sampled at the same location at each sampling campaign by scraping rocks at a depth of 15–30 cm with a sterile metal spatula (disinfected with chlorhexidine and rinsed with sterile water) [36]. Tadpoles of *A. obstetricans*, *R. temporaria,* and *B. spinosus* were swabbed for their cutaneous microbiota, one tadpole corresponding to a single microbiota sample. In the Pyrenees, *Bd* has caused mass mortality events in *A. obstetricans. R. temporaria*, and *B. spinosus* also got infected but generally at lower prevalence and mean infection burden, and without detectable mass mortalities, indicating a lower susceptibility of these two species to chytridiomycosis as compared to *A. obstetricans*, although changes in the seasonality (earlier ice thaws) may force high prevalence also in the two former species [33]. Details of amphibian skin sampling and species life traits are provided in the Supplementary material S1.

**Table 1:** Features, number and type of samples as well as *Bd* infectious status of each lakes. *Ao* = *A. obstetricans*; *Bs* = *B. spinosus*; *Rt* = *R. temporaria*; B = Biofilm; W = Water.

Site	Altitude	Coordinates	Lake area	Bd infectious status	Number of samples
Acherito (*Ach*)	1880 m	42.87; −0.70	7.46 ha	Positive	35 *Ao*; 5 *Bs;* 9 B; 12 W
Ansabère (*Ans*)	1850 m	42.88; −0.70	0.21 ha	Positive	25 *Ao*; 10 *Bs*; 10 *Rt*; 10 B; 8 W
Lhurs (*Lhu*)	1691 m	42.92; −0.70	3.59 ha	Positive	27 *Ao*; 9 B; 8 W
Puits (*Pui*)	1880 m	42.86; −0.63	0.26 ha	Positive	42 *Ao*; 9 B; 8 W
Arlet (*Arl*)	1974 m	42.84; −0.61	3.46 ha	Positive	16 *Ao*; 11 B; 8 W
Fache 1 *(FSp)*	2522 m	42.81; −0.25	0.64 ha	Negative	29 *Ao*; 3 *Rt*; 5 B; 7 W
Fache 2 *(GrF)*	2422 m	42.81; −0.25	0.87 ha	Negative	19 *Ao*; 9 B; 12 W
Embarrat *(Emb)*	2180 m	42.84; −0.19	0.01 ha	Negative	25 *Ao*; 9 *Rt*; 7 B; 6 W
Vallon *(Val)*	2215 m	42.83; −0.18	0.05 ha	Negative	33 *Ao*; 14 *Rt*; 9 B; 7 W
Paradis *(Par)*	1609 m	42.84; −0.16	0.42 ha	Unclear	25 *Ao*; 15 *Rt*; 9 B; 10 W
Madamète Haut *(MaH)*	2374 m	42.86; 0.14	0.31 ha	Unclear	28 *Rt*; 9 B; 8 W
Gourg de Rabas *(GDR)*	2400 m	42.85; 0.14	1.33 ha	Negative	7 *Ao*; 4 *Rt*; 10 B; 7 W
Pêcheur *(Pec)*	2307 m	42.86; 0.14	0.59 ha	Negative	5 *Rt*; 9 B; 8 W
Belonguère *(Bel)*	1907 m	42.84; 1.06	0.17 ha	Negative	13 *Ao*; 22 *Rt*; 12 B; 13 W
Ayès *(Aye)*	1714 m	42.84; 1.06	1.87 ha	Unclear	40 *Ao*; 15 *Rt*; 10 B; 9 W
Bethmale *(Bet)*	1063 m	42.86; 1.08	2.91 ha	Negative	5 *Bs*; 5 *Rt*; 6 B; 10 W
Labant *(Lab)*	1600 m	42.77; 1.39	0.46 ha	Negative	10 *Rt*; 9 B; 8 W
Alate *(Ala)*	1865 m	42.77; 1.40	2.13 ha	Negative	15 *Rt*; 13 B; 8 W
Lac Mort *(Mor)*	1651 m	42.76; 1.42	0.86 ha	Negative	2 *Bs*; 12 B; 7 W
Arbu *(Arb)*	1737 m	42.81; 1.43	5.01 ha	Negative	10 *Rt*; 11 B; 8 W

### 16S ribosomal ribonucleic acid gene polymerase chain reaction amplification, sequencing, and metabarcoding analyzes

Deoxyribonucleic acid (DNA) from all samples was extracted using the Macherey-Nagel™ NucleoSpin Soil kit™ (Valencia, CA, USA) according to the manufacturer protocol. Negative controls were produced from the 0.22 μm filter (*n* = 6), the DNA extraction kit (*n* = 2), and the PCR reagents (*n* = 5). The V3–V4 regions of the 16S rRNA gene was amplified by PCR using the S-D-Bact-0341-b-S-17 forward and S-D-Bact-0785-a-A-21 reverse primer described in [37], with an expected amplicon size of 464 base pairs. DNA samples were pooled in equimolar amounts in accordance with the recommended protocol for sequencing on an Illumina MiSeq system using the MiSeq Reagent Kit v2. The libraries preparation and the MiSeq Illumina sequencing of the PCR products was performed by GeTBiopuces (Toulouse, France; https://get-biopuces.insa-toulouse.fr/). Raw sequences were processed according to details given in Supplementary material S1.

### Characterization of putative anti-*Batrachochytrium dendrobatidis* bacteria

We used the anti-*Bd* database as described in [30], to detect bacterial genera known for their protective properties against *Bd* (hereafter putative anti-*Bd*). This database brings together the results of a bibliographic review of 13 articles, providing a list of 86 bacterial genera containing strains which have been shown *in vitro* to harbor *Bd* inhibitory capacities. We validated the robustness of using genera as proxy of *Bd* inhibitory bacteria as described in [30]. To this end, we analyzed the correlation between the relative abundance of putative anti-*Bd* genera, and the relative abundance of Amplicon Sequence Variants (ASVs) whose sequences match those described in the database from [38]. We performed a stand-alone BLASTN analysis on a Linux virtual machine, comparing our sequences with those in the database. We used thresholds of 100% nucleotide similarity and considered only alignments longer than 200 nucleotides [39, 40]. This showed a strong correlation (*R* = 0.70, *P* < .001), confirming the relevance of using genera as proxy for the detection of putative anti-*Bd* bacteria.

### Analysis of microbial richness and diversity of biofilm, water, and tadpole skin

We computed α-diversity (Shannon, Simpson, and Evenness) as well as β-diversity (Bray–Curtis dissimilarity distances) indexes, non-metric multidimensional scaling (NMDS), and analysis of similarities (ANOSIM) statistical analyzes using the vegan package v2.6.4. for R 4.3.2 [41]. We selected those diversity indexes because of their complementarity, the Shannon index being more sensitive to rare ASVs as compared to the Simpson index, and Evenness describing the homogeneity of the observed species distribution. Richness and diversity indexes were compared between compartment using an analysis of variance (ANOVA) followed by a Tukey test. We used linear mixed-effects models (LMM) with the *lmer* function from the lme4 R package v1.1–35.5 to explore the relationships between the α-diversity of the water and the biofilm microbiota (included in two separate models), and the α-diversity of skin microbiota. Lake and sampling year were integrated as random effects in the MLL models.

### Coalescence between environment and tadpole skin communities

The origin of the amphibian skin communities from water and biofilm was estimated using the SourceTracker software [42] set with the default parameters (rarefaction = 1000 reads; burn-in = 100; restart = 10). Computation was performed on the ASV reads independently for each lake. The SourceTracker was run three times and the confidence of the estimation was validated using the relative standard deviation (RSD) from these three runs (e.g. [43]). Wilcoxon tests were used to compare the richness index values between each compartment, as well as the percentage of contribution from water and biofilm microbial sources between *Bd*-uninfected and *Bd*-infected lakes. Results are presented as mean ± standard deviation. We also used MLL to investigate how the α-diversity of water and biofilm samples are related to the inferred percentage of environmental contribution to the amphibian skin microbiota (with lake and sampling year as random effects). To estimate probable horizontal microbial transfer between tadpoles, we compared the contributions from unknown sources, inferred by SourceTracker, between two types of lakes: those with only one amphibian species sampled (no horizontal transfer between species), and those with multiple amphibian species sampled (horizontal transfer possible). If significant but masked horizontal transfer occurred in lakes where several amphibian species were sampled, we would see an increased contribution from unknown sources. Due to the distribution of amphibian species, microbial transfers were analyzed exclusively on *Bd*-negative lakes for *R. temporaria* and *Bd*-positive lakes for *A. obstetricans*. Contribution values between the lakes with and without possible horizontal transfers were confronted using Wilcoxon tests.

We analyzed the forces driving the community assemblies (deterministic vs. stochastic) using a phylogenetic null model and computation of the Beta Nearest Taxon Index (βNTI) [5]. We used Clustal Omega to align the ASV sequences [44], and the MAFFT software to build the Neighbor Joining phylogenetic tree [45]. Phylogenetic distance between sequences were estimated with the *cophenetic* function of the Stats attached base package of the R software. We computed the observed Mean Nearest Taxon Distance (MNTD) using the relative abundance of each ASV, and the function *mntd* from the Picante package v1.8.2. The null MNTD distribution was calculated from random permutations of taxa in each community (*n* = 999). The βNTI were estimated with the formula


$$ \beta NTI=\frac{MNTD_{obs}-{MNTD}_{rand}}{SD\left({MNTD}_{rand}\right)} $$

where *MNTD_obs_* is the average distance to the nearest taxon observed in the community, *MNTD_rand_* the average of the null MNTD distances, and *SD(MNTD_rand_)* the standard deviation of null *MNTD* distances. A βNTI of zero means a fully stochastic distribution of communities, while values farther from zero indicate deterministic processes.

## Results

### Amphibian skin, biofilm, and water samples bacterial diversity

**Figure 2:**
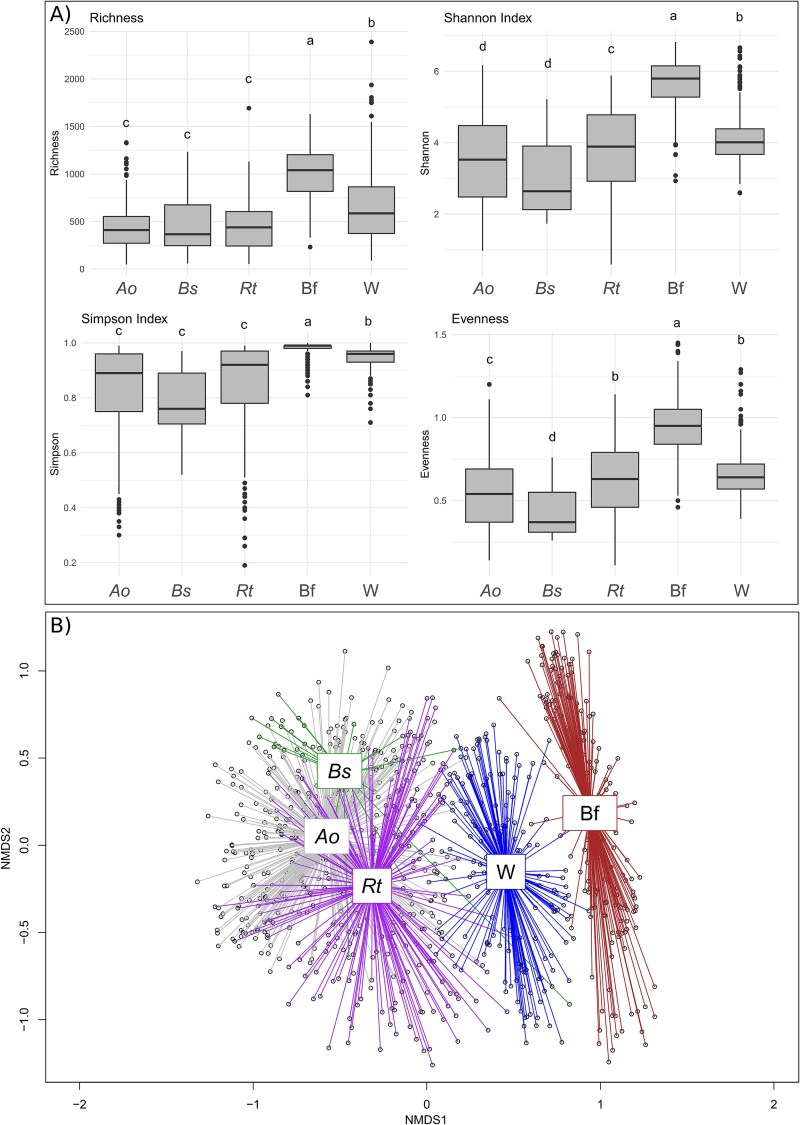
(A) Boxplot showing the richness and diversity indexes computed for the amphibian skin, biofilm and water samples. Letter indicate significant differentiation (ANOVA followed by a Tukey test). (B) NMDS representation of the Bray–Curtis dissimilarity matrix computed from the 16S rRNA gene ASV profiles of samples from *A. obstetricans* (*Ao*), *R. temporaria* (*Rt*), *B. spinosus* (*Bs*) skin, water (W) and biofilm (Bf) (stress = 0.106).

Biofilm and water diversity and richness showed significant dissimilarities, as biofilm communities exhibited the highest α-diversity on average (biofilm ASV numbers: 910 ± 333, Shannon: 5.61 ± 0.72, Simpson: 0.98 ± 0.03, Evenness: 0.84 ± 0.13, all Tukey tests *P* < .001) (Fig. 2A; Table S2). Water communities showed the second highest average diversity for ASV number (684 ± 405), Shannon (4.13 ± 0.69), Simpson (0.95 ± 0.03) indexes (all Tukey tests *P* < .05), but showed a similar Evenness in *R. temporaria* (Tukey tests *P* > .05) (Fig. 2A; Table S2). For amphibian samples, the differences were more inconsistent. Microbiota of tadpoles of all three amphibian species had equivalent ASV numbers (*A. obstetricans*: 439 ± 223, *B. spinosus*: 448 ± 297, *R. temporaria*: 439 ± 254) (all Wilcoxon tests *P* > .05, Table S2). *R. temporaria* and *A. obstetricans* showed higher diversity (Shannon 3.76 ± 1.24 and 3.45 ± 1.17, Simpson 0.84 ± 0.18 and 0.83 ± 0.15, respectively) and were more evenly distributed (0.61 ± 0.22 and 0.53 ± 0.20, respectively) than *B. spinosus* (2.97 ± 1.13, 0.78 ± 0.12 and 0.43 ± 0.16 respectively) (all Wilcoxon tests *P* < .05, except for the Shannon indexes between *A. obstetricans* and *B. spinosus*, Table S2). However, *R. temporaria* showed a significantly higher Shannon diversity index than *A. obstetricans* (*W_145_ =* 26 756, *P* = .003)*,* but not a higher Simpson index (*W_49_ =* 28 544, *P* = .063), suggesting a higher number of rare ASVs within *R. temporaria* samples.

The analysis of the β-diversity clearly showed significant segregation between the three groups formed by the tadpoles, the biofilms and the water communities (ANOSIM test, *N* = 999 permutations, R = 0.69, *P* < .01, Fig. 2B). Water communities were found to be closer to those of tadpoles than those of biofilms. Within the amphibian groups, the profiles of *R. temporaria* and *B. spinosus* microbiota were more similar (ANOSIM test, R = 0.26, *P* < .01) compared to those of *A. obstetricans* (ANOSIM test, respectively R = 0.43 and 0.51, *P* < .01). Considering only water and biofilm communities, there was a significant segregation between these two compartments (ANOSIM test, R = 0.68, *P* < .01), and also segregation within compartment according to the lake of origin (ANOSIM test, respectively R = 0.71 and 0.88, *P* < .01, Fig. S1). The *Bd* infection status of the amphibian population was not linked to the microbial assemblies of biofilm and water samples (ANOSIM, all *P* > .05), but significantly affected amphibian skin microbiota composition (ANOSIM, R = 0.05, *P* < .001).

The taxonomic affiliation of the ASVs allowed the classification of 99.88% of the reads into 152 classes and highlighted differences between the various compartments (Fig. S2). Water microbiota showed high occurrence of *γ-proteobacteria* (33.39%), *α-proteobacteria* (21.66%), and *Actinobacteria* (20.05%). The biofilm microbiota also showed high abundance of *α-proteobacteria* (21.66%) and *γ-proteobacteria* (16.21%), but was dominated by *Cyanobacteria* (27.06%) (Fig. S2). The skin microbiota of the three amphibian species showed similar patterns with high abundance of *γ-proteobacteria* (between 46.29 and 50.40%), *α-proteobacteria* (4.14 to 20.21%), *Bacteroidia* (10.06 to 16.41%), and *Clostridia* (6.47 to 16.79%).

At the genus level, the taxonomic affiliation of the ASVs allowed the classification of 78.4% of the reads into 1287 well differentiated genera. Water microbiota showed high rates of *Limnohabitans* (14.10%), but also of the Hgcl clade (14.53%) and *Flavobacterium* (6.96%). In the biofilm microbiota, abundances of the dominant genera were lower, in line with the higher diversity indexes computed. Biofilm communities were nevertheless dominated by *Cyanobium* (4.54%), an unclassified *Leptolyngbyaceae* (3.58%), and *Leptolyngbya* (2.53%). The microbiota of the three amphibian species was dominated by *Limnohabitans* (between 9.06 and 28.62%). *A. obstetricans* skin microbiota showed high rates of an unclassified *Comamonadaceae* (6.84%) and *Rhodoferax* (3.64%). *R. temporaria* skin microbiota was dominated by the putative *Bd-*inhibitory genera *Pseudomonas* (2.70%) which was less abundant in the other two amphibian species. *B. spinosus* skin samples were dominated by *Flavobacterium* (5.96%), *Dechloromonas* (2.20%), and *Bacillus* (1.58%).

### Analysis of the amphibian skin communities with biofilm and water as sources

**Figure 3:**
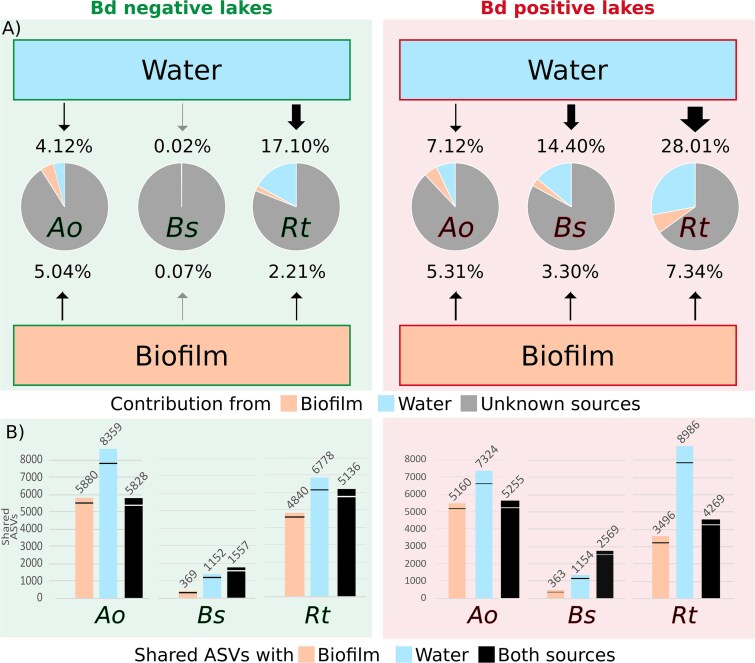
The microbial coalescence between the water or the biofilm sources, and the amphibian skin (*Ao* = *A. obstetricans*; *Bs* = *B. spinosus*, *Rt* = *R. temporaria*), compared between *Bd* positive and *Bd* negative lakes. (A) Results of the percentage of contribution. The thickness of arrows is proportional to the size of the contribution. Grey arrows indicate a contribution of <1%. (B) Numbers of shared ASVs between the three compartments. The horizontal line indicates the number of ASVs affiliated to putative anti-*Bd* genera. Scatter plot were illustrated using the ggplot2 v3.4.4 package.

**Figure 4:**
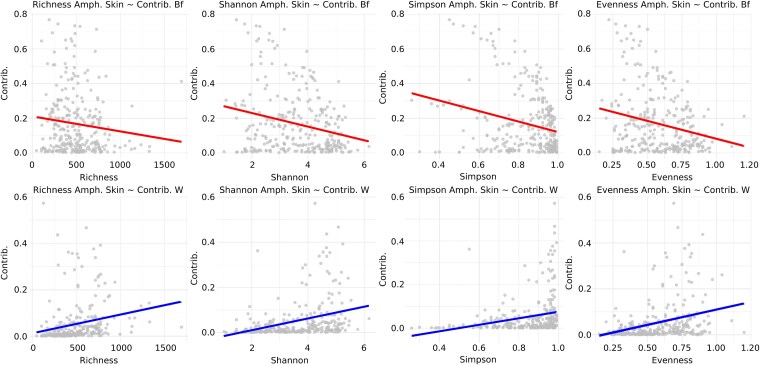
Scatter plots showing the relationship between microbial diversity metrics (richness, Shannon, Simpson, evenness) on amphibian skin and the contribution from biofilm (top row, red lines) or water (bottom row, blue lines). Each plot includes a fitted linear regression line to visualize the direction of association. Data points represent individual samples, and asterisk showed a significant *P*-value according to the LMM.

The α-diversity of amphibian skin microbiota was not linked to the α-diversity of the biofilm and water microbiota (MLL, all *P* > .05, Table S3). The Bayesian SourceTracker analysis highlighted that the amphibian skin microbiota sourced significantly from both water and biofilm (Figs. 3 and S3, Table 2). On average, environmental water contributed more than biofilm communities to skin microbiota of *A. obstetricans*, *B. spinosus,* and *R. temporaria* (*W_270_* = 75 480, *P* < .001; *W_19_* = 220, *P* < .05; *W_109_* = 5694, *P* < .001, Table S4). Water contribution to biofilm communities (7.87 ± 2.20%) was higher than its contribution to amphibian skin microbiota, except for *R. temporaria*. Due to overwintering individuals in *A. obstetricans*, we also ran SourceTracker only for this species, using the biofilm and water samples of the previous year (2016 or 2017) as sources, but the analysis did not find significant engraftment signatures. A higher α-diversity of water microbiota increased the contribution toward amphibian skin microbiota (ASVs number, Shannon, Simpson, and Evenness indexes, MLL, all *P* < .01, Fig. 4, Table S5). In contrast, the richer the biofilms, the lower its contribution toward tadpole skin microbiota (Shannon, Simpson, and Evenness indexes, MLL, all *P* < .01, Fig. 4). Analysis of the fate of the engrafted community of amphibian tadpole skin showed that stochastic rather than deterministic processes were at play during community assembly, with βNTI values of |0.71 ± 0.77| for *A. obstetricans*, |0.83 ± 0.43| for *B. spinosus* and |0.57 ± 0.55| for *R. temporaria*.

**Table 2:** Average relative contributions (± standard deviation) inferred by the SourceTracker Bayesian analysis of the ASVs from water and biofilm sources in the build-up of amphibian skin communities, according to the *Bd* infectious status of lakes. Confrontation between infected and uninfected lakes excluded Ayes, Paradis, Mort and Madamète-Haut for which the presence of *Bd* remains unclear at the time of sampling. Significantly different environmental contribution between *Bd*-negative and *Bd*-positive lakes are indicated in bold (Wilcoxon test, *P* < .05).

Lake *Bd* status	Host species	Biofilm contrib.	Water contrib.	Unknown contrib.
All	*A. obstetricans*	4.72% ± 8.64	8.85% ± 13.23	83.43% ± 17.42
	*B. spinosus*	3.04% ± 4.15	9.78% ± 14.76	87.18% ± 18.26
	*Rana temporaria*	5.81% ± 9.62	22.68% ± 19.60	71.51% ± 19.41
Positive	*A. obstetricans*	5.31% ± 8.89	**7.12% ± 13.76**	87.87% ± 13.39
	*B. spinosus*	**3.30% ± 3.02**	**14.40% ± 17.06**	82.30% ± 15.81
	*R. temporaria*	7.34% ± 1.44	**28.01% ± 11.48**	64.65% ± 12.04
Negative	*A. obstetricans*	5.04% ± 9.31	**4.12% ± 10.77**	90.84% ± 12.62
	*B. spinosus*	**0.07% ± 5.70**	**0.02% ± 2.27**	99.91% ± 0.21
	*R. temporaria*	2.21% ± 9.62	**17.10% ± 19.62**	80.69% ± 21.57

We assessed ASV sharing across amphibian skin, biofilm, and water (Fig. S3). Only 1310 ASVs were common to all compartments, while most were compartment-specific—especially in biofilm (75% of total ASVs), water (45%), and *R. temporaria* and *A. obstetricans* (both 39%). *B. spinosus* had the fewest unique ASVs (16%). A total of 26 548 ASVs were shared between at least one amphibian and the environment (Fig. S3), but this did not align with inferred environmental contributions to skin microbiota. *A. obstetricans* shared 8907 ASVs (38%) with water, 6092 (26%) with biofilm, and 5995 (26%) with both; *B. spinosus* shared 1251 (28%) with water, 411 (9%) with biofilm, and 1598 (36%) with both; and *R. temporaria* shared 9443 (40%) with water, 4913 (21%) with biofilm, and 5208 (22%) with both. At the genus level, 628 taxa were shared across all compartments, and 225 were shared between amphibians and the environment. Among 42 shared putative *Bd*-inhibitory genera (e.g. *Flavobacterium*, *Pseudomonas*), most occurred in both skin and environment, while some were exclusive to either water (*Parafrigobacterium*) or biofilm (*Nocardia*, *Kitasatospora*, and *Plantibacter*).

### Interspecific transfers of amphibian skin communities (horizontal transfer)

We analyzed the ASVs and genera shared only between the amphibian skin microbiotas in order to investigate potential horizontal transfers between hosts. The three amphibian species shared 582 ASVs of which 30 were affiliated to putative *Bd*-inhibitory genera. In addition, *A. obstetricans* shared 336 ASVs with *B. spinosus* (53 putative anti-*Bd*), and 1567 with *R. temporaria* (85 putative anti-*Bd*). *R. temporaria* and *B. spinosus* shared 163 ASVs (6 putative anti-*Bd*). These shared genera and ASVs are unlikely to result from horizontal transfers. To assess potential horizontal transfer, we compared the proportion of unknown sources, estimated by our SourceTracker analysis, for a given host species between lakes where it was the only species present (no interspecific transfer possible) and lakes with multiple sympatric amphibian species (horizontal transfer possible). For example, for *R. temporaria*, the proportion of unknown sources did not differ significantly between one-species lakes (82.85 ± 17.16%) and sympatric lakes (72.95 ± 23.17%; *W_26_* = 26.091, *P =* .458). Similarly, for *A. obstetricans*, no significant difference was observed (one-species lakes: 90.32 ± 11.77%; sympatric lakes: 84.42 ± 13.46%; W_26_ = 26.185, *P =* .453).

### Impact of the presence of *Batrachochytrium dendrobatidis* on transfers to the amphibian skin communities

Microbial sourcing from water and biofilm microbiota was analyzed in regard to the unambiguous historical presence or absence of *Bd* infections in the lake (Table 1, Figs. 3 and S5). Water communities contributed more to host microbiota in *Bd*-positive compared to *Bd*-negative lakes (*W_528_* = 23 495, *P* < .001, Tables 2 and S4, *R. temporaria*: *W_109_* = 264; *P* = .011, *B. spinosus*: *W_19_* = 0, *P* = .001, Tables 2 and S4, *A. obstetricans*: *W_270_* = 6715, *P* < .001, Fig. S5, Tables 2 and S4). We did not observe a similar pattern for biofilm microbiota as a source, except for *B. spinosus* (*W_19_* = 4, *P* < .001, Fig. 3, S5, Table 2, S4). Our phylogenetic model showed small differences according to the *Bd* infection status, with a βNTI slightly higher in *Bd*-negative lakes compared to *Bd*-positive sites, but with a high variability between amphibian individuals (*Bd*-negative vs. *Bd*-positive lakes for *A. obstetricans*: |0.79 ± 1.05| vs. |0.64 ± 0.41|; *B. spinosus*: |0.89 ± 0.73| vs. |0.79 ± 0.43|; *R. temporaria*: |0.58 ± 1.17| vs. |0.51 ± 0.26|). This suggests that stochastic processes are more dominant in *Bd*-positive lakes.

The number of ASVs shared between biofilm and/or water, and skin microbial communities was not impacted by the *Bd* infection status of the lakes (Fig. 3B, S6). In addition, the presence of *Bd* did not impact the sharing of putative anti-*Bd* bacteria between the compartments (water, biofilm, and skin) (Fig. 3B).

## Discussion

We explored the coalescence processes driving the skin microbiota assembly of three amphibian species inhabiting high mountain lakes by using water and biofilm as sources. Our results suggest a strong impact of the host population exposure to *Bd* on the microbial coalescence. In the presence of *Bd*, the transfer appears to be followed by an increased enrichment of aquatic microbes in the amphibian skin community, whereas transfers were mainly driven by passive and stochastic events. Dysbiosis and/or modification of the skin by *Bd* may select few bacteria in their new (host) habitat, or favor the proliferation of opportunistic bacteria having anti-*Bd* properties. Further, our results highlight significant microbial transfers and engraftment from the water, contributing on average 14% to the amphibian skin microbiota, while the biofilm, on which tadpoles feed, contributed only 5%.

We found that coalescent bacteria originated mainly from environmental water, and less from biofilms, and assume that this was because (i) bacteria in biofilms are part of a stable community within a protective matrix [46], and are likely less able and less ready to detach and colonize new habitats, and (ii) planktonic microorganisms are free floating and may more readily colonize new habitats [47]. Additionally, the stability of biofilms appears to be correlated to their microbial richness [48], which is in line with the negative correlation observed between the α-diversity of biofilms and the percentage of contributions from this environmental source. Around 80% of the contribution came from unknown sources, a level similar to what was observed in a previous experimental study [23]. Unknown contributions likely originated from vertical transmission from parents, as well as from environmental microbial sources that were not considered, such as sediments and macrophyte biofilms. Our results may also reflect temporal dynamics and a lag between environmental sources and host-associated communities. Although water serves as the main source, microbial colonization of the host or biofilm may occur with a delay, so that the composition of the sink community at any given time does not perfectly mirror the source. This asynchrony is consistent with the high proportion of stochastic processes observed in our analyzes. Although sampling adults could have helped to assess the role of vertical transmission more directly, this was not possible for our populations as adult amphibians disperse in terrestrial habitats, far from breeding sites, and are not necessarily related to the sampled tadpoles. Our results also suggest that, despite frequent contact when in sympatry, tadpoles of different species do not appear to exchange large amounts of bacteria. Previous studies have shown that direct horizontal transfers within a single species, although possible, tend to be less effective than indirect environmental transfers [25].

The microbial transfers and engraftment from environmental sources were found to be in line with the susceptibility of the three monitored amphibian species to *Bd* infection and chytridiomycosis, with *A. obstetricans* recruiting on average half as many bacteria as *R. temporaria* and *B. spinosus*. Biofilm and water of the previous year did not make an apparent contribution to the skin microbiota of overwintering tadpoles of *A. obstetricans*. Our observation underlines the importance of the contribution of other environmental microbial sources, e.g. contributions from the biological father and the terrestrial environment while carrying the eggs [49], or from sediments during winter (for overwintering tadpoles). An early acquisition of microbes at the egg stage and complemented during winter, could then make colonization by other bacteria more difficult due to inhibitory priority effects [50]. Priority effects have already been determined as a major factor influencing the coalescence mechanisms [51]. These points could explain the lower relative abundance of *Bd*-inhibitory genera observed within *A. obstetricans* skin microbiota [30], for whom it may be more difficult to settle in the engrafted community, contributing to explaining the greater susceptibility of this species to *Bd*.

The presence of *Bd* in the population was found to influence the microbial transfers from water microbial source. Considering the contribution of water to biofilm communities (~7%) as a proxy for baseline passive microbial transfer, the lower contribution observed on amphibian skin in *Bd*-negative populations indicates effective host-mediated filtering of environmental microorganisms. In contrast, the increased water contribution to skin microbiota under *Bd* infection suggests an active selection or enrichment of specific environmental taxa, rather than a simple passive colonization process. It is known that *Bd* can cause major shifts in amphibian skin microbiota [52], leading to dysbiosis [53]. A healthy microbiome is more resilient to external invasion and therefore less likely to recruit new microbial cells [54], limiting opportunities for opportunistic or pathogenic colonizers. Other studies have suggested that anti-*Bd* bacteria can be recruited during *Bd*-induced community shifts [29]. Our results further suggest that environmental sources, and particularly water, play a central role in these assembly processes. It is to be noted that we used the proxy from Loyau and colleagues [30] to identify putative anti-*Bd* bacteria, acknowledging its limitations: functional traits can vary within genera, and *in vitro* inhibition may not reflect in situ effectiveness due to ecological and host-specific factors. The approach, however, revealed a consistent pattern.

The apparent increase in water-derived bacterial contribution in *Bd*-positive populations was not associated with a higher number of shared ASVs between the environment and the host. The environmental influence, hence, does not reflect a greater diversity or quantity of microbial acquisition per se, but rather a selective enrichment of certain taxa on the host skin, reflecting engraftment [5]. This is further supported by the different microbial assemblies observed on the skin of infected versus uninfected amphibian populations, while biofilm and water microbial communities remained unaffected by the presence of *Bd*, suggesting that amphibians have access to similar environmental microbial seeding sources, but selectively filter and enrich specific bacteria depending on the host infectious status and immune condition. Our study echoes findings from studies on other amphibian pathogens, such as ranaviruses, where environmental microbial richness was also linked to increased host resistance [55]. Finally, the comparable number of anti-*Bd* ASVs shared between skin, biofilms, and water in both *Bd*-positive and *Bd*-negative populations suggests that infection status does not drive a specific, targeted recruitment of protective taxa, but may instead favor the selective retention and proliferation of certain environmental bacteria already present in richer microbial pools.

The dysbiosis caused by *Bd* infection could be at the origin of stochastic assemblies, as dysbiosis increases the ecological dispersal and drift [52], as well as the colonization effect by environmental opportunistic microorganisms [56]. Stochastic assemblies have been shown to dominate after a disturbance whereas it takes a long time for deterministic processes to take hold [57]. The differentiated living conditions resulting from *Bd* colonization of the skin may be to the detriment of specialist bacteria, and may profit some environmental opportunistic bacteria [58]. Environmental opportunistic bacteria are more adapted to colonizing new niches due to their rapid growth rate, their resistance capacity or their use of a wide variety of energy sources [59], including acquisition of nutrients from fungal cells for some *Pseudomonas* strains [60]. According to a previous study, in the presence of *Bd*, the most enriched putative *Bd*-inhibitory genera were *Flavobacterium*, *Acinetobacter*, *Aeromonas*, *Bacillus*, *Hafnia*, *Novosphingobium*, and *Pseudomonas* [30], which were shown to be environmental opportunists [61–63]. Environmental opportunistic bacteria could remain in a dormant state through dormancy or sporulation mechanisms [59], and emerge in the event of changes [61] such as a (re-)infection by a pathogen [15]. These mechanisms are in line with the adaptative microbiome hypothesis suggesting the selection of bacteria that could benefit the amphibian host and increase resilience to the *Bd* pathogen [15], a process that takes time.

Our study provides important insights for a better understanding of coalescence phenomena that shape the amphibian skin microbiota in natural settings, when exposed to *Bd*. Exposure to *Bd* was linked to an enrichment of microorganisms from the environment in the engrafted communities, including some environmental opportunistic bacteria. Our results indicate that *Bd* presence influences bacterial enrichment, not recruitment, in amphibian skin microbiota. Post-transfer, community assembly appeared stochastic, potentially favoring opportunistic microbes that help resist *Bd* infection. Colonization by opportunistic bacteria have already been documented in other host species after disturbances such as changes in coral mucus [64], sponge exposed to antibiotics [56] or rats after burn injury [65], but also in abiotic compartments such as deglaciated soils [66] or soils exposed to high temperature [67], suggesting similar re-assembly processes after disturbance of all sorts.

## Supplementary Material

VF_Bouchali_et_al_Supp_files_ISMEcom_R1

VF_Rayan_bouchali_Linking_supp_mat_S1_ycag016

## Data Availability

The datasets generated and/or analysed during the current study are available in the Inventaire des Données de la Recherche et Environnement et Sociétés (InDoRES) repository, 10.48579/PRO/2OMFAG (private link for reviewer: https://data.indores.fr:443/privateurl.xhtml?token=66f5f6eb-e39b-4906-9230-a2cd992c8cf9).
