# Supplementary material for: The fungal pathogen Batrachochytrium dendrobatidis drives the relationship between environmental and amphibian skin microbiota
Source: ISME Commun. 2026 Feb 4;6(1):ycag016. doi: 10.1093/ismeco/ycag016 (PMC12927882; doi:10.1093/ismeco/ycag016)
Supplement: VF_Bouchali_et_al_Supp_files_ISMEcom_R1 [file vf_bouchali_et_al_supp_files_ismecom_r1.docx]

**The fungal pathogen *Batrachochytrium dendrobatidis* drives the relationship between environmental and amphibian skin microbiota**

Rayan Bouchali^1*^, Hugo Sentenac^2^, Dirk S. Schmeller^1^, Adriana Bernardo-Cravo^1^, Adeline Loyau^1^

^1^Université de Toulouse, Toulouse INP, CNRS, IRD, CRBE, Toulouse, France

^2^Université Marie et Louis Pasteur, CNRS, Chrono-environnement (UMR 6249), F-25000 Besançon, France

*Corresponding author: rayan.bouchali@toulouse-inp.fr

**
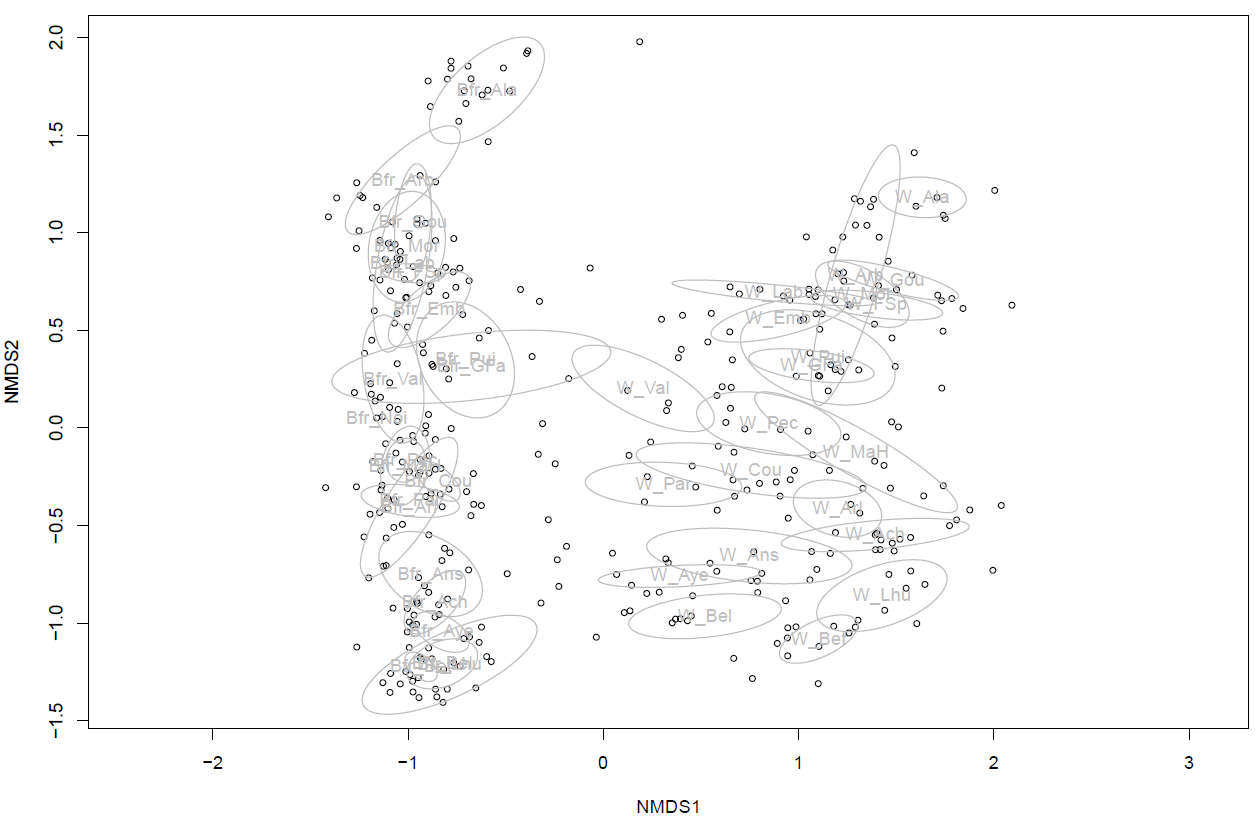
Fig. S1.** Non-metric multidimensional scaling (NMDS) representation of the Bray-Curtis dissimilarity matrix computed from the 16S rRNA gene ASV profiles (**Table S1**) of samples from water (W) and biofilms (B) according to their lake of origin (stress = 0.07).

**Fig. S2.** Stacked bar charts showing the average relative abundance of the 15 more represented bacterial classes (relative abundance superior to 0.5%). Bacterial classes are indicated on the right and are ranked in descending order of abundance.


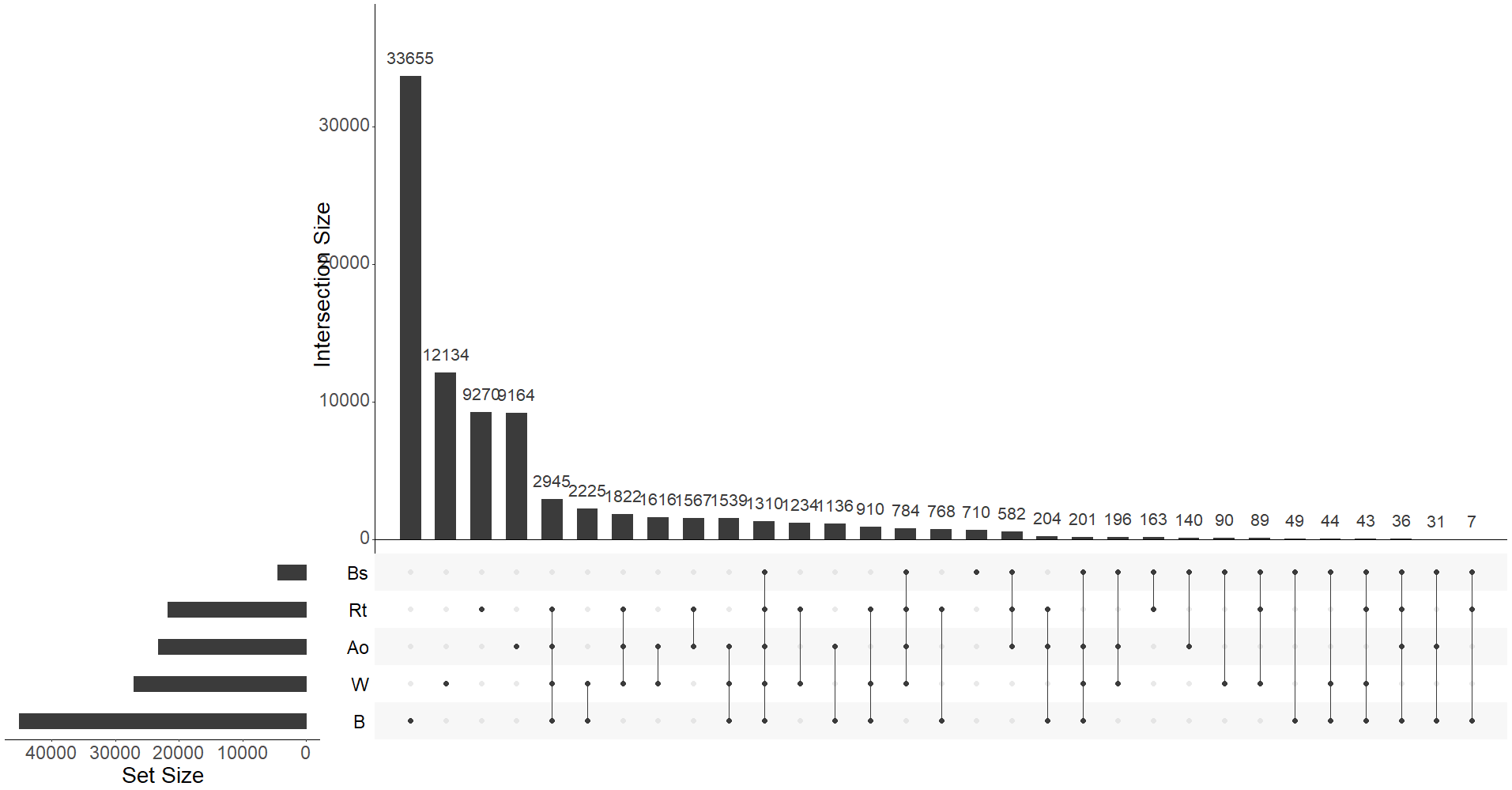


**Fig. S3.** Intersection plot showing the repartition of the 16S rRNA ASVs between the five compartments for all the lakes. Ao = *A. obstetricans*, Bs = *B. spinosus*, Rt = *R. temporaria*, B = Biofilm and W = Water. Intersection plot was computed on R using the UpSetR package v1.4.0 (Conway et al., 2017).


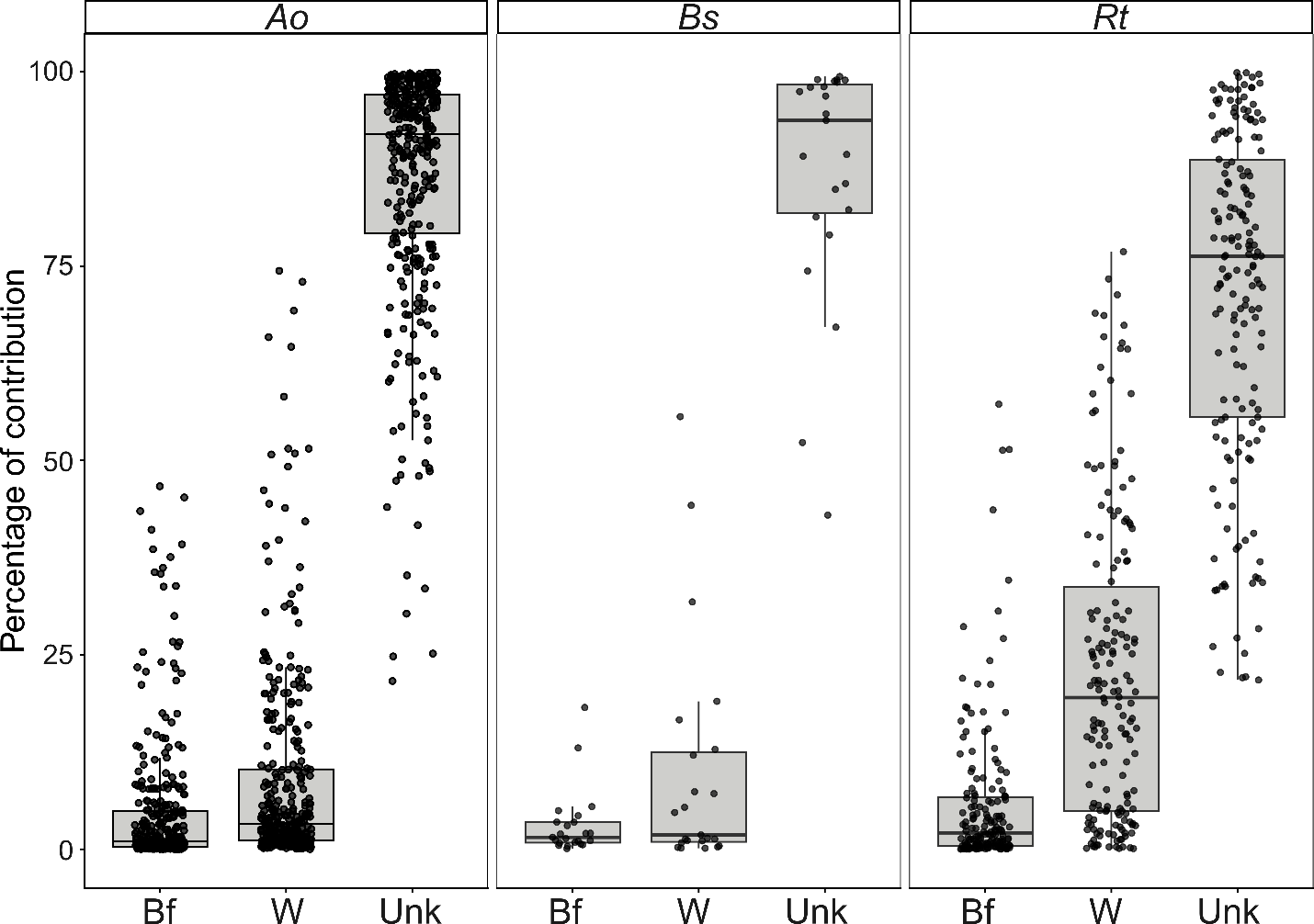


**Fig. S4.** Boxplots showing the percentage of contribution from biofilm (Bf) water (W) sources, as well as unknown sources (Unk), to the amphibian skin bacterial communities. *Ao* = *A. obstetricans*; *Bs* = *B. spinosus*, *Rt* = *R. temporaria*. Boxplots were visualized using the ggplot2 v 3.4.4 package (Wickham, 2016).


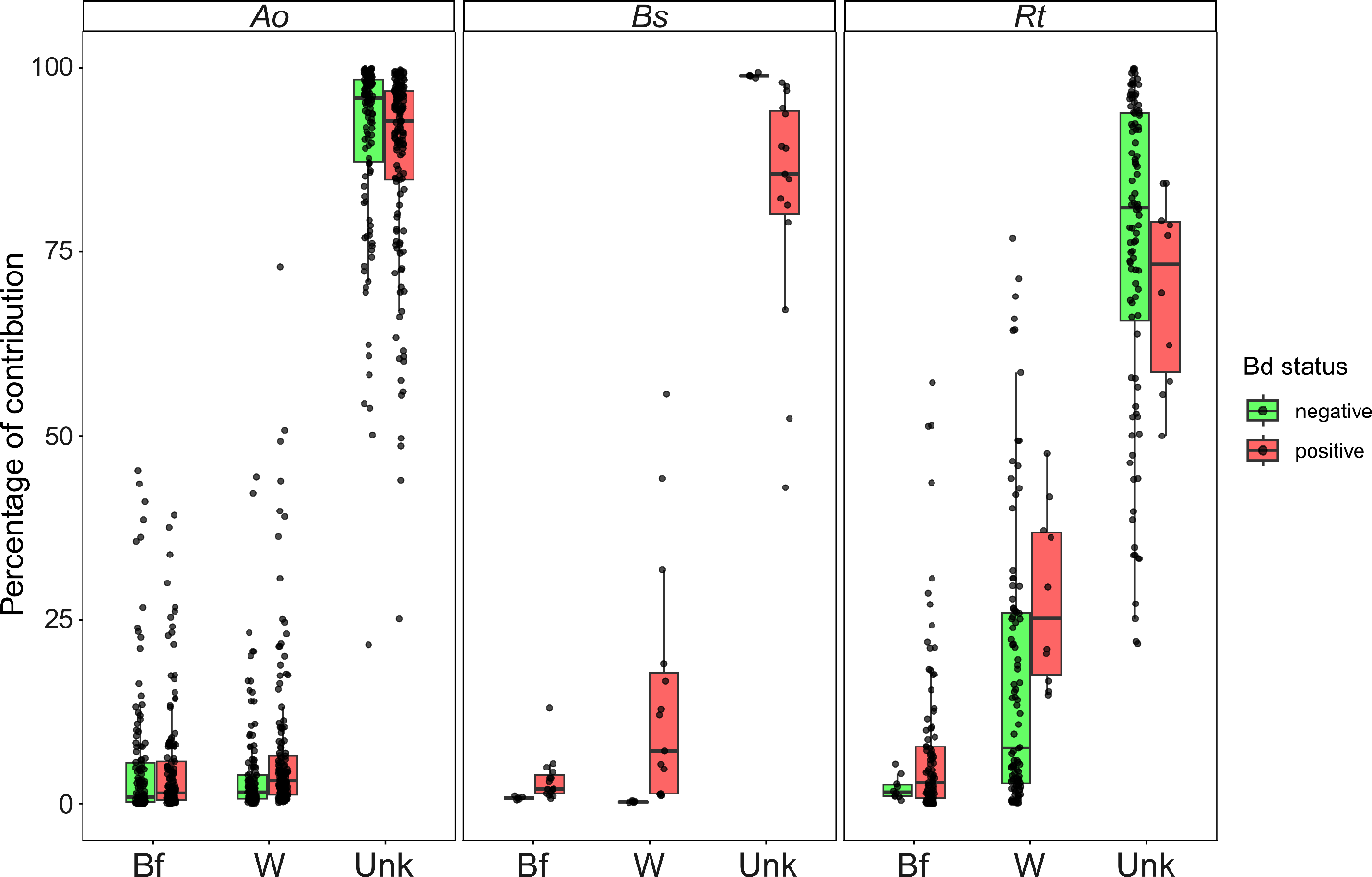


**Fig. S5.** Boxplots showing the percentage of contribution from biofilm (Bf) water (W) sources, as well as unknown sources (Unk), to the amphibian skin bacterial communities, according to the *Bd* infectious status. *Ao* = *A. obstetricans*; *Bs* = *B. spinosus*, *Rt* = *R. temporaria*. Boxplots were visualized using the ggplot2 v 3.4.4 package (Wickham, 2016).


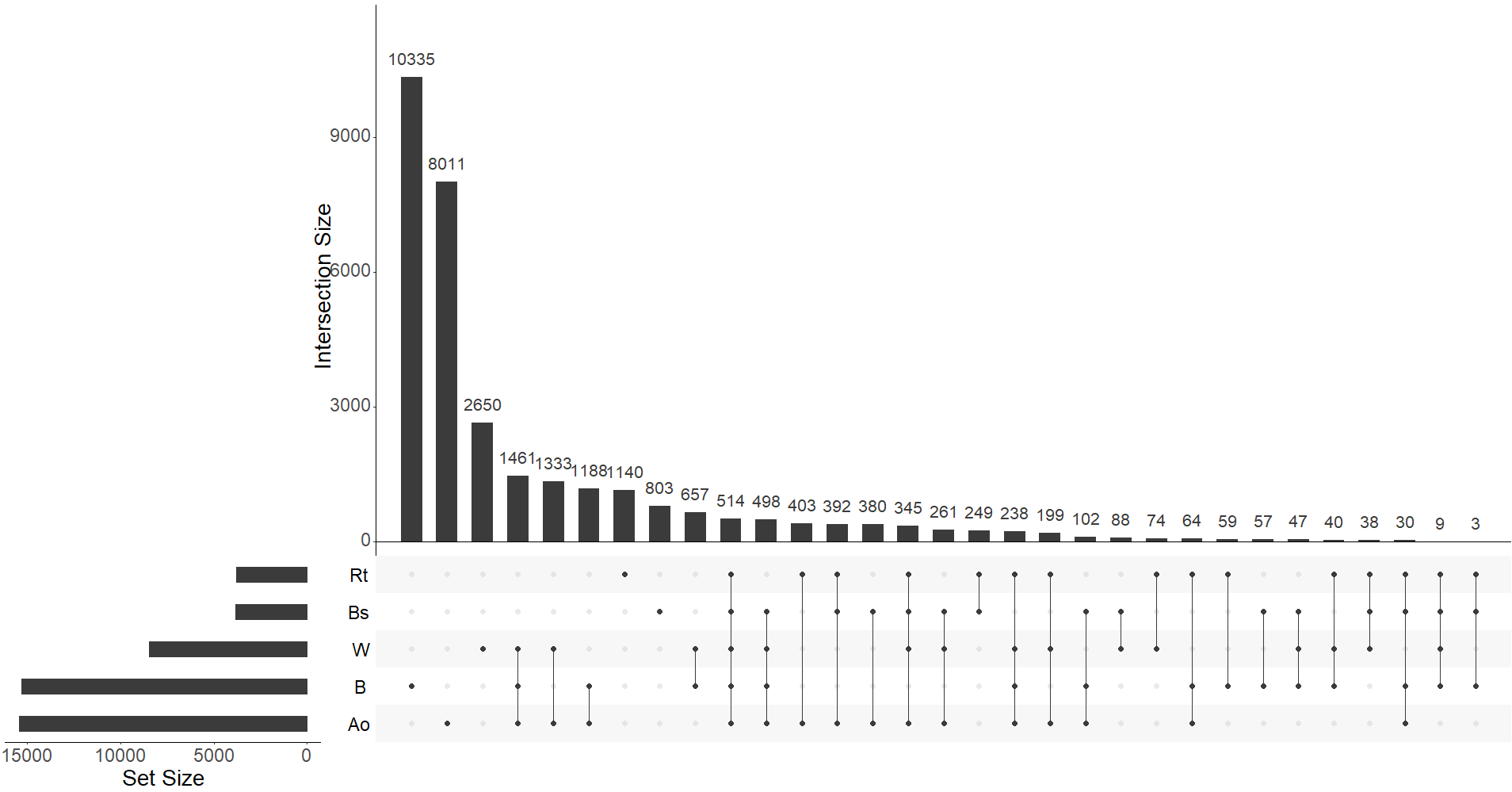


A)


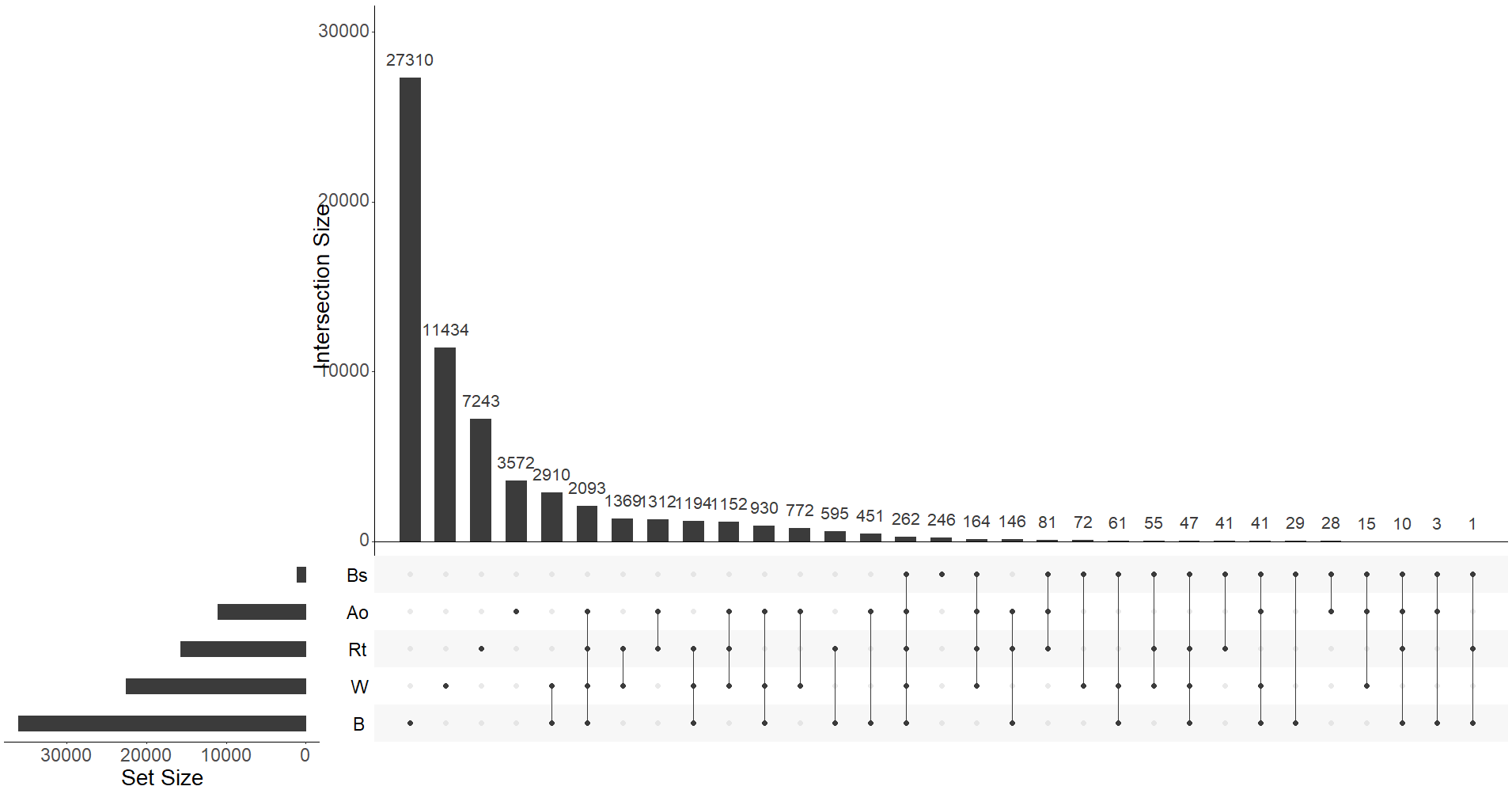


B)

**Fig. S6.** Intersection plot showing the repartition of the 16S rRNA ASVs between the five compartments for A) the Bd positive lakes and B) the Bd negative lakes. Ao = *A. obstetricans*, Bs = *B. spinosus*, Rt = *R. temporaria*, B = Biofilm and W = Water. Intersection plots were computed on R using the UpSetR package v1.4.0 (Conway et al., 2017).

**Table S1.** Sampling year, host species, developmental stage, number of samples, prevalence, mean *Bd* load (in genome equivalents, GE), and standard error of the mean (SEM) for Pyrenean mountain lakes, listed from west to east.

| **Year** | **Site** | **Species** | **Developmental Stage** | **N** | **Prevalence (%)** | **Mean *Bd* load (GE)** | **SEM *Bd* load (GE)** |
| --- | --- | --- | --- | --- | --- | --- | --- |
| 2016 | Acherito | *Alytes obstetricans* | Metamorph | 30 | 53 | 66.31 | 28.09 |
| 2017 | Acherito | *Alytes obstetricans* | Metamorph | 21 | 19 | 1.41 | 0.89 |
| 2017 | Acherito | *Alytes obstetricans* | Tadpole | 63 | 67 | 319.65 | 65.32 |
| 2017 | Acherito | *Bufo spinosus* | Metamorph | 10 | 10 | 8.25 | – |
| 2018 | Acherito | *Alytes obstetricans* | Metamorph | 20 | 50 | 3.88 | 1.72 |
| 2018 | Acherito | *Alytes obstetricans* | Tadpole | 70 | 56 | 29.19 | 9.37 |
| 2016 | Ansabère | *Alytes obstetricans* | Metamorph | 20 | 75 | 14.74 | 7.37 |
| 2016 | Ansabère | *Alytes obstetricans* | Tadpole | 16 | 69 | 197.06 | 88.04 |
| 2016 | Ansabère | *Bufo spinosus* | Metamorph | 30 | 10 | 1.07 | 0.84 |
| 2016 | Ansabère | *Rana temporaria* | Metamorph | 29 | 0 |  |  |
| 2017 | Ansabère | *Alytes obstetricans* | Tadpole | 42 | 0 |  |  |
| 2017 | Ansabère | *Bufo spinosus* | Metamorph | 15 | 0 |  |  |
| 2017 | Ansabère | *Rana temporaria* | Metamorph | 3 | 0 |  |  |
| 2018 | Ansabère | *Alytes obstetricans* | Tadpole | 36 | 22 | 21.00 | 10.74 |
| 2016 | Lhurs (Lake) | *Alytes obstetricans* | Metamorph | 29 | 48 | 40.73 | 15.50 |
| 2016 | Lhurs (Lake) | *Alytes obstetricans* | Tadpole | 30 | 80 | 121.89 | 33.17 |
| 2016 | Lhurs (Lake) | *Bufo bufo* | Tadpole | 30 | 3 | 0.26 | – |
| 2017 | Lhurs (Lake) | *Alytes obstetricans* | Metamorph | 31 | 35 | 0.71 | 0.36 |
| 2017 | Lhurs (Lake) | *Alytes obstetricans* | Tadpole | 96 | 28 | 239.15 | 68.33 |
| 2017 | Lhurs (Lake) | *Bufo spinosus* | Metamorph | 17 | 6 | 0.18 | – |
| 2018 | Lhurs (Lake) | *Alytes obstetricans* | Metamorph | 15 | 33 | 1.65 | 0.74 |
| 2018 | Lhurs (Lake) | *Alytes obstetricans* | Tadpole | 60 | 12 | 18.07 | 16.80 |
| 2018 | Lhurs (Lake) | *Rana temporaria* | Metamorph | 15 | 0 |  |  |
| 2016 | Puits | *Alytes obstetricans* | Metamorph | 14 | 100 | 53.31 | 18.43 |
| 2016 | Puits | *Alytes obstetricans* | Tadpole | 25 | 64 | 210.97 | 50.24 |
| 2017 | Puits | *Alytes obstetricans* | Metamorph | 18 | 67 | 51.07 | 28.89 |
| 2017 | Puits | *Alytes obstetricans* | Tadpole | 37 | 78 | 1162.25 | 351.70 |
| 2018 | Puits | *Alytes obstetricans* | Metamorph | 20 | 90 | 481.75 | 413.65 |
| 2018 | Puits | *Alytes obstetricans* | Tadpole | 43 | 16 | 169.31 | 65.51 |
| 2016 | Arlet | *Alytes obstetricans* | Metamorph | 30 | 53 | 3295.75 | 810.13 |
| 2016 | Arlet | *Alytes obstetricans* | tadpole | 40 | 100 | 110.59 | 29.88 |
| 2016 | Arlet | *Rana temporaria* | Metamorph | 29 | 0 |  |  |
| 2017 | Arlet | *Alytes obstetricans* | Metamorph | 26 | 100 | 373.20 | 206.57 |
| 2017 | Arlet | *Alytes obstetricans* | Tadpole | 30 | 100 | 480.55 | 86.31 |
| 2017 | Arlet | *Bufo spinosus* | Metamorph | 15 | 60 | 77.62 | 71.93 |
| 2017 | Arlet | *Rana temporaria* | Metamorph | 19 | 53 | 0.88 | 0.16 |
| 2018 | Arlet | *Alytes obstetricans* | Metamorph | 30 | 60 | 143.46 | 105.55 |
| 2018 | Arlet | *Alytes obstetricans* | Tadpole | 11 | 64 | 81.38 | 29.02 |
| 2018 | Arlet | *Rana temporaria* | Metamorph | 16 | 0 |  |  |
| 2016 | Fache Esp. | *Alytes obstetricans* | Tadpole | 10 | 0 |  |  |
| 2017 | Fache Esp. | *Alytes obstetricans* | Tadpole | 40 | 0 |  |  |
| 2018 | Fache Esp. | *Alytes obstetricans* | Tadpole | 30 | 0 |  |  |
| 2016 | Grand Fache | *Alytes obstetricans* | Tadpole | 30 | 0 |  |  |
| 2017 | Grand Fache | *Alytes obstetricans* | Tadpole | 35 | 0 |  |  |
| 2018 | Grand Fache | *Alytes obstetricans* | Tadpole | 30 | 0 |  |  |
| 2017 | Embarrat | *Alytes obstetricans* | Tadpole | 30 | 0 |  |  |
| 2018 | Embarrat | *Alytes obstetricans* | Tadpole | 29 | 0 |  |  |
| 2016 | Vallon | *Alytes obstetricans* | Tadpole | 30 | 0 |  |  |
| 2017 | Vallon | *Alytes obstetricans* | Tadpole | 30 | 0 |  |  |
| 2018 | Vallon | *Alytes obstetricans* | Tadpole | 30 | 0 |  |  |
| 2016 | Paradis | *Alytes obstetricans* | Tadpole | 30 | 0 |  |  |
| 2017 | Paradis | *Alytes obstetricans* | Tadpole | 15 | 7 | 0.26 | – |
| 2018 | Paradis | *Alytes obstetricans* | Tadpole | 32 | 13 | 0.37 | 0.02 |
| 2018 | Gourg de Rabas | *Alytes obstetricans* | Tadpole | 16 | 0 |  |  |
| 2016 | Belonguère | *Alytes obstetricans* | Tadpole | 30 | 0 |  |  |
| 2017 | Belonguère | *Alytes obstetricans* | Tadpole | 9 | 0 |  |  |
| 2018 | Belonguère | *Alytes obstetricans* | Tadpole | 3 | 0 |  |  |
| 2017 | Ayès | *Alytes obstetricans* | Tadpole | 30 | 0 |  |  |
| 2018 | Ayès | *Alytes obstetricans* | Tadpole | 30 | 50 | 40.65 | 13.11 |
| 2018 | Alate | *Rana temporaria* | Tadpole | 10 | 0 |  |  |
| 2018 | Lac Mort | *Rana temporaria* | Tadpole | 10 | 0 |  |  |
| 2016 | Arbu | *Alytes obstetricans* | Tadpole | 31 | 0 |  |  |

**Table S2.** Results of the Wilcoxon test computed on the diversity indices infered from the 16S rRNA gene ASVs between each compartments. Benjamini-Hochberg correction was performed to compute adjusted p-value. “*” shows p-value <0.05.

| **Diversity indices tested** | **Test** | **Statistics** | | | | | |
| --- | --- | --- | --- | --- | --- | --- | --- |
| **Richness** | **ANOVA** | **Source** | **Df** | **Sum Sq** | **Mean Sq** | **F value** | **Pr(>F)** |
|  |  | group | 4 | 56,676,526 | 14,169,132 | 163.9 | <0.001* |
|  |  | Residuals | 1,027 | 88,778,208 | 86,444 |  |  |
|  | **Tukey HSD** | **Comparison** | **diff** | **lwr** | **upr** | **p adj** |  |
|  |  | Bs - Ao | 8.510 | -164.560 | 181.580 | 1.000 |  |
|  |  | Rt - Ao | 0.280 | -73.050 | 73.600 | 1 |  |
|  |  | Bf - Ao | 569.810 | 503.110 | 636.510 | <0.001* |  |
|  |  | W - Ao | 239.770 | 171.260 | 308.280 | <0.001* |  |
|  |  | Rt - Bs | -8.230 | -185.870 | 169.410 | 1.000 |  |
|  |  | Bf - Bs | 561.300 | 386.300 | 736.310 | <0.001* |  |
|  |  | W - Bs | 231.260 | 55.560 | 406.960 | <0.001* |  |
|  |  | Bf - Rt | 569.530 | 491.740 | 647.320 | <0.001* |  |
|  |  | W - Rt | 239.490 | 160.150 | 318.840 | <0.001* |  |
|  |  | W - Bf | -330.040 | -403.310 | -256.770 | <0.001* |  |
| **Shannon** | **ANOVA** | **Source** | **Df** | **Sum Sq** | **Mean Sq** | **F value** | **Pr(>F)** |
|  |  | group | 4 | 792.1 | 198 | 197.1 | <0.001* |
|  |  | Residuals | 1,027 | 1,031.7 | 1 |  |  |
|  | **Tukey HSD** | **Comparison** | **diff** | **lwr** | **upr** | **p adj** |  |
|  |  | Bs - Ao | -0.481 | -1.071 | 0.109 | 0.170 |  |
|  |  | Rt - Ao | 0.307 | 0.057 | 0.557 | 0.007* |  |
|  |  | Bf - Ao | 2.197 | 1.970 | 2.424 | <0.001* |  |
|  |  | W - Ao | 0.682 | 0.449 | 0.916 | <0.001* |  |
|  |  | Rt - Bs | 0.788 | 0.182 | 1.393 | 0.004* |  |
|  |  | Bf - Bs | 2.678 | 2.081 | 3.275 | <0.001* |  |
|  |  | W - Bs | 1.163 | 0.564 | 1.762 | <0.001* |  |
|  |  | Bf - Rt | 1.891 | 1.625 | 2.156 | <0.001* |  |
|  |  | W - Rt | 0.376 | 0.105 | 0.646 | 0.002* |  |
|  |  | W - Bf | -1.515 | -1.765 | -1.265 | <0.001* |  |
| **Simpson** | **ANOVA** | **Source** | **Df** | **Sum Sq** | **Mean Sq** | **F value** | **Pr(>F)** |
|  |  | group | 4 | 4.727 | 1.182 | 79.6 | <0.001* |
|  |  | Residuals | 1,027 | 15.248 | 0.015 |  |  |
|  | **Tukey HSD** | **Comparison** | **diff** | **lwr** | **upr** | **p adj** |  |
|  |  | Bs - Ao | -0.051 | -0.122 | 0.021 | 0.301 |  |
|  |  | Rt - Ao | 0.011 | -0.019 | 0.042 | 0.842 |  |
|  |  | Bf - Ao | 0.150 | 0.122 | 0.177 | <0.001* |  |
|  |  | W - Ao | 0.118 | 0.089 | 0.146 | <0.001* |  |
|  |  | Rt - Bs | 0.062 | -0.011 | 0.136 | 0.143 |  |
|  |  | Bf - Bs | 0.200 | 0.128 | 0.273 | <0.001* |  |
|  |  | W - Bs | 0.168 | 0.096 | 0.241 | <0.001* |  |
|  |  | Bf - Rt | 0.138 | 0.106 | 0.171 | <0.001* |  |
|  |  | W - Rt | 0.106 | 0.073 | 0.139 | <0.001* |  |
|  |  | W - Bf | -0.032 | -0.062 | -0.002 | 0.032* |  |
| **Evenness** | **ANOVA** | **Source** | **Df** | **Sum Sq** | **Mean Sq** | **F value** | **Pr(>F)** |
|  |  | group | 4 | 27.97 | 6.992 | 196.2 | <0.001* |
|  |  | Residuals | 1,027 | 36.6 | 0.036 |  |  |
|  | **Tukey HSD** | **Comparison** | **diff** | **lwr** | **upr** | **p adj** |  |
|  |  | Bs - Ao | -0.106 | -0.217 | 0.005 | 0.071 |  |
|  |  | Rt - Ao | 0.080 | 0.033 | 0.127 | <0.001* |  |
|  |  | Bf - Ao | 0.416 | 0.373 | 0.459 | <0.001* |  |
|  |  | W - Ao | 0.131 | 0.087 | 0.175 | <0.001* |  |
|  |  | Rt - Bs | 0.186 | 0.072 | 0.300 | <0.001* |  |
|  |  | Bf - Bs | 0.522 | 0.410 | 0.634 | <0.001* |  |
|  |  | W - Bs | 0.237 | 0.124 | 0.350 | <0.001* |  |
|  |  | Bf - Rt | 0.336 | 0.286 | 0.386 | <0.001* |  |
|  |  | W - Rt | 0.051 | 0.000 | 0.102 | 0.052 |  |
|  |  | W - Bf | -0.285 | -0.332 | -0.238 | <0.001* |  |

**Table S3.** Results of the linear mixed-effects model computed between the diversity indices from amphibian skin samples, and those from environmental (biofilm and water) ones. Benjamini-Hochberg correction was performed to compute adjusted p-value. “*” shows p-value <0.05. *Ao* = *A. obstetricans*, *Bs* = *B. spinosus*, *Rt* = *R. temporaria*, Bf = biofilm and W = water.

| **model** | **term** | **estimate** | **std.error** | **conf.low** | **conf.high** | **p.value** | **Sig.** |
| --- | --- | --- | --- | --- | --- | --- | --- |
| Richness Ao ~ Bf | (Intercept) | 487.32 | 101.89 | 278.48 | 696.15 | <0.001 | * |
| Richness Ao ~ Bf | richness_bf | -0.08 | 0.09 | -0.26 | 0.11 | 0.404 |  |
| Richness Ao ~ W | (Intercept) | 303.16 | 76.86 | 130.37 | 475.94 | 0.003 | * |
| Richness Ao ~ W | richness_w | 0.12 | 0.07 | -0.02 | 0.26 | 0.089 |  |
| Shannon Ao ~ Bf | (Intercept) | 5.08 | 1.04 | 2.97 | 7.18 | <0.001 | * |
| Shannon Ao ~ Bf | shannon_bf | -0.31 | 0.18 | -0.68 | 0.07 | 0.104 |  |
| Shannon Ao ~ W | (Intercept) | 4.45 | 0.62 | 3.20 | 5.70 | <0.001 | * |
| Shannon Ao ~ W | shannon_w | -0.26 | 0.14 | -0.54 | 0.02 | 0.072 |  |
| Simpson Ao ~ Bf | (Intercept) | 1.86 | 0.63 | 0.59 | 3.14 | 0.005 | * |
| Simpson Ao ~ Bf | simpson_bf | -1.07 | 0.65 | -2.37 | 0.24 | 0.106 |  |
| Simpson Ao ~ W | (Intercept) | 1.16 | 0.42 | 0.31 | 2.01 | 0.009 | * |
| Simpson Ao ~ W | simpson_w | -0.36 | 0.44 | -1.25 | 0.54 | 0.426 |  |
| Evenness Ao ~ Bf | (Intercept) | 0.72 | 0.14 | 0.44 | 1.00 | <0.001 | * |
| Evenness Ao ~ Bf | evenness_bf | -0.21 | 0.14 | -0.49 | 0.08 | 0.152 |  |
| Evenness Ao ~ W | (Intercept) | 0.64 | 0.08 | 0.48 | 0.81 | <0.001 | * |
| Evenness Ao ~ W | evenness_w | -0.19 | 0.11 | -0.40 | 0.03 | 0.084 |  |
| Richness Bs ~ Bf | (Intercept) | 1,628.50 | 861.99 | -2,080.33 | 5,337.34 | 0.199 |  |
| Richness Bs ~ Bf | richness_bf | -1.28 | 0.85 | -4.92 | 2.36 | 0.270 |  |
| Richness Bs ~ W | (Intercept) | 299.46 | 448.66 | -1,630.98 | 2,229.91 | 0.573 |  |
| Richness Bs ~ W | richness_w | 0.06 | 0.59 | -2.46 | 2.59 | 0.923 |  |
| Shannon Bs ~ Bf | (Intercept) | 5.79 | 10.06 | -37.49 | 49.08 | 0.623 |  |
| Shannon Bs ~ Bf | shannon_bf | -0.51 | 1.74 | -8.01 | 6.99 | 0.797 |  |
| Shannon Bs ~ W | (Intercept) | 0.07 | 2.08 | -8.87 | 9.00 | 0.977 |  |
| Shannon Bs ~ W | shannon_w | 0.59 | 0.48 | -1.47 | 2.65 | 0.341 |  |
| Simpson Bs ~ Bf | (Intercept) | -2.63 | 4.19 | -20.65 | 15.39 | 0.594 |  |
| Simpson Bs ~ Bf | simpson_bf | 3.47 | 4.25 | -14.83 | 21.76 | 0.501 |  |
| Simpson Bs ~ W | (Intercept) | -0.92 | 0.94 | -5.40 | 3.56 | 0.439 |  |
| Simpson Bs ~ W | simpson_w | 1.74 | 0.97 | -2.91 | 6.40 | 0.229 |  |
| Evenness Bs ~ Bf | (Intercept) | 1.05 | 1.33 | -4.69 | 6.78 | 0.514 |  |
| Evenness Bs ~ Bf | evenness_bf | -0.73 | 1.53 | -7.29 | 5.84 | 0.680 |  |
| Evenness Bs ~ W | (Intercept) | -0.07 | 0.54 | -2.38 | 2.24 | 0.903 |  |
| Evenness Bs ~ W | evenness_w | 0.65 | 0.76 | -2.62 | 3.91 | 0.483 |  |
| Richness Rt ~ Bf | (Intercept) | 493.93 | 124.70 | 234.92 | 752.94 | 0.001 | * |
| Richness Rt ~ Bf | richness_bf | 0.01 | 0.12 | -0.25 | 0.27 | 0.917 |  |
| Richness Rt ~ W | (Intercept) | 495.13 | 79.97 | 331.32 | 658.94 | <0.001 | * |
| Richness Rt ~ W | richness_w | -0.06 | 0.07 | -0.21 | 0.09 | 0.431 |  |
| Shannon Rt ~ Bf | (Intercept) | 2.87 | 1.00 | 0.78 | 4.96 | 0.009 | * |
| Shannon Rt ~ Bf | shannon_bf | 0.24 | 0.18 | -0.13 | 0.62 | 0.191 |  |
| Shannon Rt ~ W | (Intercept) | 3.22 | 0.79 | 1.60 | 4.85 | 0.000 | * |
| Shannon Rt ~ W | shannon_w | 0.18 | 0.19 | -0.21 | 0.57 | 0.345 |  |
| Simpson Rt ~ Bf | (Intercept) | -0.28 | 0.76 | -1.86 | 1.30 | 0.716 |  |
| Simpson Rt ~ Bf | simpson_bf | 1.21 | 0.78 | -0.41 | 2.82 | 0.136 |  |
| Simpson Rt ~ W | (Intercept) | 0.27 | 0.70 | -1.17 | 1.71 | 0.702 |  |
| Simpson Rt ~ W | simpson_w | 0.63 | 0.75 | -0.90 | 2.15 | 0.409 |  |
| Evenness Rt ~ Bf | (Intercept) | 0.44 | 0.16 | 0.11 | 0.78 | 0.012 | * |
| Evenness Rt ~ Bf | evenness_bf | 0.25 | 0.16 | -0.09 | 0.60 | 0.137 |  |
| Evenness Rt ~ W | (Intercept) | 0.46 | 0.13 | 0.19 | 0.73 | 0.002 | * |
| Evenness Rt ~ W | evenness_w | 0.27 | 0.19 | -0.12 | 0.65 | 0.165 |  |

**Table S4.** Results of the Wilcoxon test computed on the percentage of explanation infered from the SourceTracker analyzes between each compartments and in relation with the *Bd* infectious status of lakes (“*Bd +*” and “*Bd* -”). Benjamini-Hochberg correction was performed to compute adjusted p-value. “*” shows p-value <0.05. *Ao* = *A. obstetricans*, *Bs* = *B. spinosus* and *Rt* = *R. temporaria*.

| **Host** | **Compartments tested** | **Degree of Freedom** | **W value** | **Adjusted p-value** | **Significance** |
| --- | --- | --- | --- | --- | --- |
| All hosts | Water ~ Biofilm | 528 | 83,518 | <2e-16 | * |
|  | Water *Bd* + ~ *Bd* - | 498 | 23,495 | <2e-16 | * |
|  | Biofilm *Bd* + ~ *Bd* - | 159 | 19,048 | 0.610 |  |
| *Ao* | Water ~ Biofilm | 270 | 75,480 | 3.93e-14 | * |
|  | Water *Bd* + ~ *Bd* - | 270 | 6,715 | <2e-16 | * |
|  | Biofilm *Bd* + ~ *Bd* - | 270 | 8,089 | 0.095 |  |
| *Bs* | Water ~ Biofilm | 19 | 220 | 0.042 | * |
|  | Water *Bd* + ~ *Bd* - | 19 | 0 | 0.001 | * |
|  | Biofilm *Bd* + ~ *Bd* - | 19 | 4 | 1.5e-3 | * |
| *Rt* | Water ~ Biofilm | 109 | 5,694 | <2.2e-16 | * |
|  | Water *Bd* + ~ *Bd* - | 109 | 264 | 0.011 | * |
|  | Biofilm *Bd* + ~ *Bd* - | 109 | 588 | 0.360 |  |

**Table S5.** Results of the linear mixed-effects model computed between the richness and diversity indices of amphibian skin samples (“Amph. Skin”) and the percentage of explanation (“Contrib.”) infered by the SourceTracker analysis. Benjamini-Hochberg correction was performed to compute adjusted p-value. Significance code for *p-value*: “*” shows p-value <0.05. Contrib. = infered percentage of explanation infered by the SourceTracker analysis, Bf = biofilm and W = water.

| **model** | **estimate** | **std.error** | **conf.low** | **conf.high** | **p.value** | **Sig.** |
| --- | --- | --- | --- | --- | --- | --- |
| Evenness Amph. Skin ~ Contrib. Bf | -0.32 | 0.07 | -0.46 | -0.19 | <0.001 | * |
| Evenness Amph. Skin ~ Contrib. W | 0.62 | 0.12 | 0.39 | 0.85 | <0.001 | * |
| Richness Amph. Skin ~ Contrib. Bf | -85.71 | 86.47 | -256.05 | 84.62 | 0.323 |  |
| Richness Amph. Skin ~ Contrib. W | 408.92 | 149.42 | 114.82 | 703.02 | 0.007 | * |
| Shannon Amph. Skin ~ Contrib. Bf | -1.98 | 0.40 | -2.77 | -1.18 | <0.001 | * |
| Shannon Amph. Skin ~ Contrib. W | 3.79 | 0.67 | 2.46 | 5.11 | <0.001 | * |
| Simpson Amph. Skin ~ Contrib. Bf | -0.25 | 0.06 | -0.36 | -0.13 | <0.001 | * |
| Simpson Amph. Skin ~ Contrib. W | 0.62 | 0.12 | 0.39 | 0.85 | <0.001 | * |
