# Supplementary material for: The fungal pathogen Batrachochytrium dendrobatidis drives the relationship between environmental and amphibian skin microbiota
Source: ISME Commun. 2026 Feb 4;6(1):ycag016. doi: 10.1093/ismeco/ycag016 (PMC12927882; doi:10.1093/ismeco/ycag016)
Supplement: VF_Rayan_bouchali_Linking_supp_mat_S1_ycag016 [file vf_rayan_bouchali_linking_supp_mat_s1_ycag016.docx]

**The fungal pathogen *Batrachochytrium dendrobatidis* drives the relationship between environmental and amphibian skin microbiota**

Rayan Bouchali^1*^, Hugo Sentenac^2^, Dirk S. Schmeller^1^, Adriana Bernardo-Cravo^1,^ Adeline Loyau^1^

^1^Université de Toulouse, Toulouse INP, CNRS, IRD, CRBE, Toulouse, France

^2^Université Marie et Louis Pasteur, CNRS, Chrono-environnement (UMR 6249), F-25000 Besançon, France

*Corresponding author: rayan.bouchali@toulouse-inp.fr

This file provide supplementary information on the material and method related to the manuscript **The fungal pathogen *Batrachochytrium dendrobatidis* drives the relationship between environmental and amphibian skin microbiota**

***Amphibian species life traits***

The three amphibian species show different life history traits and reproductive phenology, likely to influence the microbial transfers between their skin microbiota and the surrounding environment. *B. spinosus* and *R. temporaria* clutches are deposited directly in the water, while *A. obstetricans* eggs are first carried by the biological father. In mountain populations, *A. obstetricans* tadpoles can hibernate in lake water for several years, while *R. temporaria* and *B. spinosus* metamorphose in their birth year. *A. obstetricans* overwintered tadpoles are likely to be a *Bd* reservoir [1] because *Bd* infection is restricted to their mouthparts, and thus chytridiomycosis is non-lethal for them as opposed to metamorphosed individuals [2, 3].

***Sample of amphibian skin samples***

Individuals were captured in the littoral of the lakes by dip-netting and their skin was swabbed over their entire body using a sterile swab (MW100, MWE Medical Wire, Corsham, UK), then released immediately. All the samples were immediately frozen on dry ice (-78°C) in the field. Sampling campaigns yielded 188 biofilms and 172 water samples (**Table 1**). Not all amphibian species were present at all sites: tadpoles of *A. obstetricans* were sampled across 13 lakes (in 2016, 2017 and 2018), *R. temporaria* in 15 lakes (only in 2017 and 2018) and 3 lakes showed *B. spinosus* populations (only in 2018). A total of 552 amphibian tadpoles were sampled: 336 *A. obstetricans*, 164 *R. temporaria* and 25 *B. spinosus* (**Table 1**).

***DNA Amplification and sequencing processes***

Quality of all DNA extracts was checked using a Nanodrop ND-1000 spectrophotometer (Nanodrop Technologies LLCTM).

The V3-V4 regions of the 16S rRNA gene was amplified by PCR using the S-D-Bact-0341-b-S-17 forward (5′-CCTACGGGNGGCWGCAG-3’) and S-D-Bact-0785-a-A-21 reverse primer (5′-GACTACHVGGGTATCTAATCC-3’) described in [4]. The PCR cycling was 3 min at 95°C, 30 cycles of 30s at 55°C and 30s at 72°C, and a final extension step of 5 min at 72 °C. Quality and purity of PCR amplicons were checked on 1.5% agarose gel.

Gene primers, linkers and barcodes were removed using Cutadapt V4.0 with Python 3.9.12 [5]. Raw reads were processed with the DADA2 software v1.26.0 [6] using the Standard Operating Procedure for paired-end reads (<https://benjjneb.github.io/dada2/bigdata_paired.html>). Quality and filtering parameters used were minLen=c(200, 200), runcLen=c(280, 260), maxN=0, maxEE=c(5, 5) and truncQ=2. The *consensus* method from the *removeBineraDenovo* function was used to detect and discard chimeric sequences. Amplicon Sequence Variants (ASVs) were affiliated with the Silva v138.1 database and the *assignTaxonomy* function, using the Wang naive classifier method [7] and a minimal bootstrap of 80 %. Contaminant ASVs were discarded with the Decontam package for R [8] using the prevalence method and a threshold of 0.5. ASVs affiliated to chloroplast or mitochondria were discarded. Thus, the sequencing of the PCR amplicons allowed the generation of 87,722,690 raw reads. Processing of the amplicons by the DADA2 pipeline led to 41,627,948 clean reads. Samples Bs_Mor_1_L65_16S_2018 and Bs_Mor_1_L66_16S_2018 were removed because of a too low number of reads (respectively 0 and 39 reads). The Decontam package allowed the detection of 384 contaminant ASVs which were discarded. The final dataset comprised 36,164,412 16S rRNA gene reads distributed into 73,698 ASVs.

**References**

1. Clare FC et al. Climate forcing of an emerging pathogenic fungus across a montane multi-host community. *Philos Trans R Soc B Biol Sci* 2016;**371**:20150454. https://doi.org/10.1098/rstb.2015.0454

2. Berger L et al. Chytridiomycosis causes amphibian mortality associated with population declines in the rain forests of Australia and Central America. *Proc Natl Acad Sci* 1998;**95**:9031–9036. https://doi.org/10.1073/pnas.95.15.9031

3. Garner TWJ et al. Life history tradeoffs influence mortality associated with the amphibian pathogen *Batrachochytrium dendrobatidis*. *Oikos* 2009;**118**:783–791. https://doi.org/10.1111/j.1600-0706.2008.17202.x

4. Klindworth A et al. Evaluation of general 16S ribosomal RNA gene PCR primers for classical and next-generation sequencing-based diversity studies. *Nucleic Acids Res* 2013;**41**:e1–e1. https://doi.org/10.1093/nar/gks808

5. Martin M. Cutadapt removes adapter sequences from high-throughput sequencing reads. *EMBnet.journal* 2011;**17**:10. https://doi.org/10.14806/ej.17.1.200

6. Callahan BJ et al. DADA2: High-resolution sample inference from Illumina amplicon data. *Nat Methods* 2016;**13**:581–583. https://doi.org/10.1038/nmeth.3869

7. Wang Q et al. Naïve Bayesian Classifier for Rapid Assignment of rRNA Sequences into the New Bacterial Taxonomy. *Appl Environ Microbiol* 2007;**73**:5261–5267. https://doi.org/10.1128/AEM.00062-07

8. Davis NM et al. Simple statistical identification and removal of contaminant sequences in marker-gene and metagenomics data. *Microbiome* 2018;**6**. https://doi.org/10.1186/s40168-018-0605-2
